# Supplementary material for: The genome sequence of the Dusky Grizzled Skipper, Pyrgus cacaliae (Rambur, 1839) (Lepidoptera: Hesperiidae)
Source: Wellcome Open Res. 2025 Aug 11;10:424. [Version 1] doi: 10.12688/wellcomeopenres.24672.1 (PMC12457891; doi:10.12688/wellcomeopenres.24672.1)
Supplement: Peer review history

# Reviewer report: Reviewer response for version 1

**DOI:** 10.21956/wellcomeopenres.27187.r129621

**Published:** 2025-12-29

**Authors:** Yu-Feng Huang

**Affiliations:** 1 Computer Science and Engineering, Yuan Ze University (Ringgold ID: 34895), Zhongli District, Taoyuan City, Taiwan

This manuscript presents a chromosome-level, haplotype-resolved genome assembly of *Pyrgus cacaliae*, the Dusky Grizzled Skipper, generated under the Wellcome Sanger Institute’s Tree of Life (ToL) and Project Psyche programs. The assembly achieves excellent metrics - scaffold N50 of 26.69 Mb, BUSCO completeness of 98.6%, and QV > 64 - meeting Earth BioGenome Project (EBP) reference genome standards (6.C.Q64).

 The paper is concise, methodologically rigorous, and consistent with ToL data note conventions. It provides a high-quality reference genome for *Pyrgus*, with clear relevance for comparative Lepidopteran genomics and conservation studies. The sequencing, assembly, and curation pipelines follow standardized and well-documented workflows, ensuring long-term reproducibility.

 The manuscript is technically sound and well written. However, several clarifications would further strengthen completeness and transparency.

**Major Comments**

**1. Haplotype Representation and Explanation**

 The genome includes two haplotypes - haplotype 1 assembled to chromosome level and haplotype 2 assembled to scaffold level - yet the manuscript does not explain the basis for this difference in assembly contiguity. Please clarify whether the reduced continuity and completeness of haplotype 2 (N50 = 26.79 Mb; k-mer completeness = 74.41%) reflects biological heterozygosity, reduced read coverage, or curation thresholds applied during assembly polishing. A short explanation would help readers interpret the relative reliability and intended usage of each haplotype.

**2. Mitochondrial Genome Description**

 The mitochondrial genome (15.43 kb) is mentioned only briefly. It should be explicitly stated whether the mitogenome is complete and circularized, as circularity is a key quality criterion in mitochondrial assemblies. Providing a succinct annotation summary (e.g., 13 PCGs, 22 tRNAs, 2 rRNAs) and any comparison with existing *Pyrgus* mitogenomes would strengthen this section.

 Additionally, an INSDC accession number for the mitochondrial genome is missing and should be included.

**3. Assembly Curation Transparency**

 The authors report 11 breaks and 43 joins during manual curation, but do not specify whether these occurred at the scaffold or chromosome level. Clarifying the type, rationale, and impact of these corrections (e.g., resolving chimeric joins, removing haplotypic duplications) would enhance transparency.

 Furthermore, given that the rapid curation protocol was used, the authors should briefly indicate whether any evaluation method was applied to assess potential missing or misassembled regions following curation.

**4. BUSCO and k-mer Completeness Discussion**

 Although BUSCO completeness (98.6%) and combined k-mer completeness (99.11%) are excellent, the substantial differences between haplotypes (80.82% vs. 74.41%) warrant explanation. A short interpretative note regarding heterozygosity, haplotype collapse, or assembly limitations would assist users in understanding genome representation and completeness.

**5. Numerical or Reporting Consistency**

 There is a discrepancy between the main text and Table 4: the text reports that “94.63% of the assembly sequence was assigned to chromosomes,” while Table 4 lists “99.87%.” This inconsistency should be corrected for clarity and accuracy.

**Minor Comments**

**1. Expression of Units**

 Replace “15.43 kb” with the exact value (e.g., 15,431 bp or 15,439 bp) when referring to the mitochondrial genome. Given the small size of mitogenomes, reporting precise base pair length is preferable to rounded kilobase values.

Are sufficient details of methods and materials provided to allow replication by others?

Yes

Is the rationale for creating the dataset(s) clearly described?

Yes

Are the datasets clearly presented in a useable and accessible format?

Yes

Are the protocols appropriate and is the work technically sound?

Yes

Reviewer Expertise:

Bioinformatics, Mitogenome, Machine learning, Cancer Biology

I confirm that I have read this submission and believe that I have an appropriate level of expertise to confirm that it is of an acceptable scientific standard, however I have significant reservations, as outlined above.

# Reviewer report: Reviewer response for version 1

**DOI:** 10.21956/wellcomeopenres.27187.r129625

**Published:** 2025-09-23

**Authors:** Annabel Whibley

**Affiliations:** 1 Grapevine Improvement, BRI, Lincoln, New Zealand

In this Data Note, Chittaro and colleagues report the genome sequence of the Dusky Grizzled Skipper, *Pyrgus cacaliae. *Sequenced as part of project Psyche, the introduction sets out the natural history of this species and identifies some knowledge gaps that a genome sequence may help to address.

 Nearly 95% of the assembly could be scaffolded into 31 pseudomolecules, and the use of a female specimen means that both Z and W chromosomes are reported in this assembly, although the resolution of the W is hampered by its highly repetitive nature. The sequencing and assembly pipeline, and subsequent evaluations, follow standard DToL procedures and templating. The low-input library prep route was used.  The assembly quality is excellent, with a QV >64 and very complete BUSCO and k-mer statistics. Public data accessions are working.

 Minor comments that should be addressed: In the Genome sequence report section, the statement about coverage for the assembly backbone is little ambiguous (“HiFi sequence data” rather than  “sequencing data” would be clearer).  I am a little sceptical that the in vivo photograph in Figure 1 is the same individual as in the voucher panel below, but perhaps the legend to this figure could specify. There is also, I think, an error in the last row of Table 4. Both the abstract and main text state 94.63% of the assembly sequence is assigned to chromosomes, whereas this table states “99.87”).

Are sufficient details of methods and materials provided to allow replication by others?

Yes

Is the rationale for creating the dataset(s) clearly described?

Yes

Are the datasets clearly presented in a useable and accessible format?

Yes

Are the protocols appropriate and is the work technically sound?

Yes

Reviewer Expertise:

Genomics, bioinformatics

I confirm that I have read this submission and believe that I have an appropriate level of expertise to confirm that it is of an acceptable scientific standard.

# Reviewer report: Reviewer response for version 1

**DOI:** 10.21956/wellcomeopenres.27187.r129626

**Published:** 2025-09-11

**Authors:** Arun Arumugaperumal

**Affiliations:** 1 Department of Biotechnology, Rajalakshmi Engineering College, Chennai, Tamil Nadu, India

The data note describes the genome sequencing of a butterfly species *Pyrgus cacaliae*. The butterfly specimen has been neatly documented as a photograph in figure 1. This is the first report of the high-quality genome sequence of the organism. The assembly presented is of size 808.81 Mb spread along 31 chromosomes. The butterfly follows ZW sex system and both chromosomes were scaffolded since this was a female specimen. The mitogenome has also been sequenced which is of size 15.43 kb. High-end sequencing technology and standard protocols were used to arrive at the high-quality genome of this butterfly. There are several other Pyrgus genomes sequenced as part of Darwin tree of life and has been published. The genome size of *P. cacaliae* is found to be comparable to those genome sizes. The BUSCO completeness of 98.6% indicates that the genome sequence provided is a near-complete one. The links for accessing the data have been provided except for the Ensembl annotation. The article can be indexed.

Are sufficient details of methods and materials provided to allow replication by others?

Yes

Is the rationale for creating the dataset(s) clearly described?

Yes

Are the datasets clearly presented in a useable and accessible format?

Yes

Are the protocols appropriate and is the work technically sound?

Yes

Reviewer Expertise:

Bioinformatics; Genomics

I confirm that I have read this submission and believe that I have an appropriate level of expertise to confirm that it is of an acceptable scientific standard.
